# Changes in cerebral function parameters in persons with HIV with symptoms of insomnia switching from dolutegravir- to bictegravir-based antiretroviral therapy

**DOI:** 10.1007/s13365-025-01270-x

**Published:** 2025-08-20

**Authors:** Merle Henderson, Kate Alford, Samira Bouyagoub, Nicki Doyle, Sriram Vundavalli, Pedro Vicente, Albert Busza, Alan Winston, Jaime H. Vera

**Affiliations:** 1 Department of Infectious Disease, Faculty of Medicine, Imperial College London, London, UK; 2 Jefferiss Wing, St. Mary’s Hospital, Imperial College Healthcare NHS Trust, London, UK; 3 Department of Global Health and Infection, Brighton and Sussex Medical School, Brighton, UK; 4 University Hospital Sussex NHS Foundation Trust, Brighton, UK; 5 Clinical Imaging Sciences Centre, University of Sussex, Brighton, UK; 6 Department of Brain Sciences, Imperial College London, London, UK

**Keywords:** Antiretroviral therapy, HIV, Neuropsychiatric, Insomnia, MRI

## Abstract

Sleep disturbances are frequently reported in persons with HIV and have been associated with the use of certain integrase strand transfer inhibitors (INSTIs), such as dolutegravir. This exploratory study assessed changes in cerebral function parameters in individuals with insomnia switching INSTIs. Individuals with an insomnia severity index (ISI) above 8 and virologically suppressed on a dolutegravir-containing ART regimen (DTG-ART) were randomised 1:1 to either continue DTG-ART or switch to bictegravir/emtricitabine/tenofovir alafenamide (BIC-ART) for 120 days. Cerebral function parameters were measured longitudinally at baseline (D0) and day 120 (D120) and included: (1) patient-reported outcomes (PROs) assessing sleep, quality of life (QoL) and symptoms related to ART, (2) resting-state functional cerebral MRI (fMRI), examining functional connectivity networks previously associated with DTG use or sleep and (3) plasma soluble inflammatory biomarkers associated with neuroinflammation or HIV disease progression (Neopterin, CXCL10 and IL-6). Functional connectivity analyses were performed using Seed-Based Correlations (SBC), and correlations between connectivity changes, PRO measures and biomarker concentrations determined. Of 19 individuals (12 DTG-ART, 7 BIC-ART), median age was 55 years (range 28–83), all were male and 17 of white ethnicity. Over 120 days, improvements in sleep and QoL in those randomised to BIC-ART vs. DTG-ART were observed. Median change in Insomnia Severity Index (ISI) score − 9 (-14 to -2) vs. -1 (-10 to -4), *p* = 0.030, Epworth Sleepiness Scale (ESS) -3.0 (-6 to -1) vs. 2 (-3 to 6), *p* = 0.007 and Short Form-36 Physical Function (SF36-PF) -5 (-40 to 5) vs. 0 (-5 to 15), *p* = 0.026) for BIC- vs. DTG- ART, respectively. BIC-ART was also associated with increased functional connectivity in the Default Mode and Salience Networks (both *p* < 0.05), which correlated with improvements in PRO measures (ESS and SF36-PF, both *p* < 0.05). No significant changes in soluble biomarkers were observed. Individuals with insomnia switching to BIC-ART had improvements in self-reported sleep, QoL and resting state fMRI networks associated with sleep, when compared to those continued on DTG-ART.

## Introduction

With access to widespread effective antiretroviral therapy (ART) there has been a decline in the incidence of AIDS-related central nervous system (CNS) conditions in persons with HIV (Garvey et al., 2011). Despite this, neuropsychiatric symptoms such as, anxiety, depression, and sleep disruption, remain common (Rezaei et al., 2019; Brandt et al., 2017; Kunisaki et al., 2021) and may significantly impact on health-related quality of life (Kunisaki et al., 2021; Brandt et al., 2017).

While the aetiology of these symptoms may be multifactorial, specific ART agents and combinations may play a contributory role. Historically, neurotoxicity associated with the non-nucleoside reverse transcriptase inhibitor (NNRTI) efavirenz has been well documented (Mollan et al., 2014; Ciccarelli et al., 2011; Staszewski et al., 1999). In more recent years, accumulating data from diverse cohorts have indicated an association between the integrase-strand transfer inhibitors (INSTI) and neuropsychiatric side effects, particularly insomnia and sleep disorders (Hoffmann and Llibre 2019; de Boer et al., 2016). These INSTI-related side effects have been demonstrated in both persons with HIV commencing ART for the first time and in those on ART switching ART regimens, with greater discontinuation rates observed with dolutegravir, when compared to other INSTIs such as bictegravir, raltegravir or cabotegravir (Hoffmann and Llibre 2019; de Boer et al., 2016; Nasreddine et al., 2020; Raffi et al., 2013). The rates of dolutegravir discontinuation are highly variable within clinical cohort studies ranging between 2 and 15%, possibly highlighting differences in awareness, diagnosis and management of neuropsychiatric symptoms across different settings (de Boer et al., 2016; Nasreddine et al., 2020; Raffi et al., 2013; Hill et al., 2018; Cabello-Úbeda et al., 2022). While previous data have suggested a potential additive role of abacavir, a nucleoside reverse transcriptase inhibitor (NRTI), in contributing toward sleep disturbances in persons with HIV on dolutegravir (Hoffmann et al., 2017), more recent data report an increased frequency of sleep disturbances with dolutegravir independent of abacavir use, when compared to other non-dolutegravir-containing ART-regimens (Osiyemi et al., 2022).

Clinical studies investigating neuropsychiatric symptoms among persons with HIV rely heavily on subjective measures, notably the collection of self-reported symptoms through questionnaires. While these methods are valuable, they do not offer insights into the potential mechanisms underlying the symptoms. Objective measures, such as Blood Oxygen Level Dependent (BOLD) resting state functional magnetic resonance imaging (fMRI), explore how different brain regions interact with each other and possess the capability to discern variations in brain activation induced by different pharmacological agents (Borsook et al., 2013; Toniolo et al., 2020). Employing such objective measures enables a more comprehensive understanding of the potential mechanisms responsible for the observed symptoms.

Using fMRI we have previously demonstrated changes in cerebral functional connectivity in persons with HIV with viral suppression without neuropsychiatric symptoms switching from raltegravir- to dolutegravir-containing ART (Toniolo et al., 2020). Current data suggest bictegravir-containing ART (BIC-ART) may have fewer central nervous system side effects compared to dolutegravir-containing ART (DTG-ART) (Wohl et al., 2019). To our knowledge, no data exists comparing functional connectivity measured by fMRI in persons with HIV with neuropsychiatric symptoms switching from DTG-ART to BIC-ART. We aimed to compare longitudinal changes in cerebral function parameters, measured by fMRI, patient reported outcomes and plasma inflammatory biomarkers of brain health, in persons with suppressed HIV and symptoms of insomnia, switching from DTG- to BIC-ART.

## Methods

### Study design and participants

This pilot study was an open label, randomised controlled trial which enrolled at two UK-based study sites (Imperial College Healthcare NHS Trust, London and University Sussex Hospital NHS Foundation Trust, Brighton) between October 2021 and January 2023. Inclusion criteria were persons with HIV aged 18 years or older with evidence of insomnia, defined as an insomnia severity index score of 8 or more, who were virologically suppressed (plasma HIV RNA concentrations < 50 copies/mL) on DTG-ART for a minimum of 4 months. Exclusion criteria included those with evidence of HIV drug-resistance mutations, previous exposure to bictegravir, significant neurological disease, major depression or psychosis, a diagnosed sleep disorder, use of medication which could interfere with sleep, substance misuse, active opportunistic infection, significant comorbidities or pregnancy. All participants provided written informed consent. Ethics approval was obtained from the UK Research Ethics Committee (20/SC/0116). Study database registration; EudraCT 2019-004007-12.

### Study procedures

**Fig. 1:**
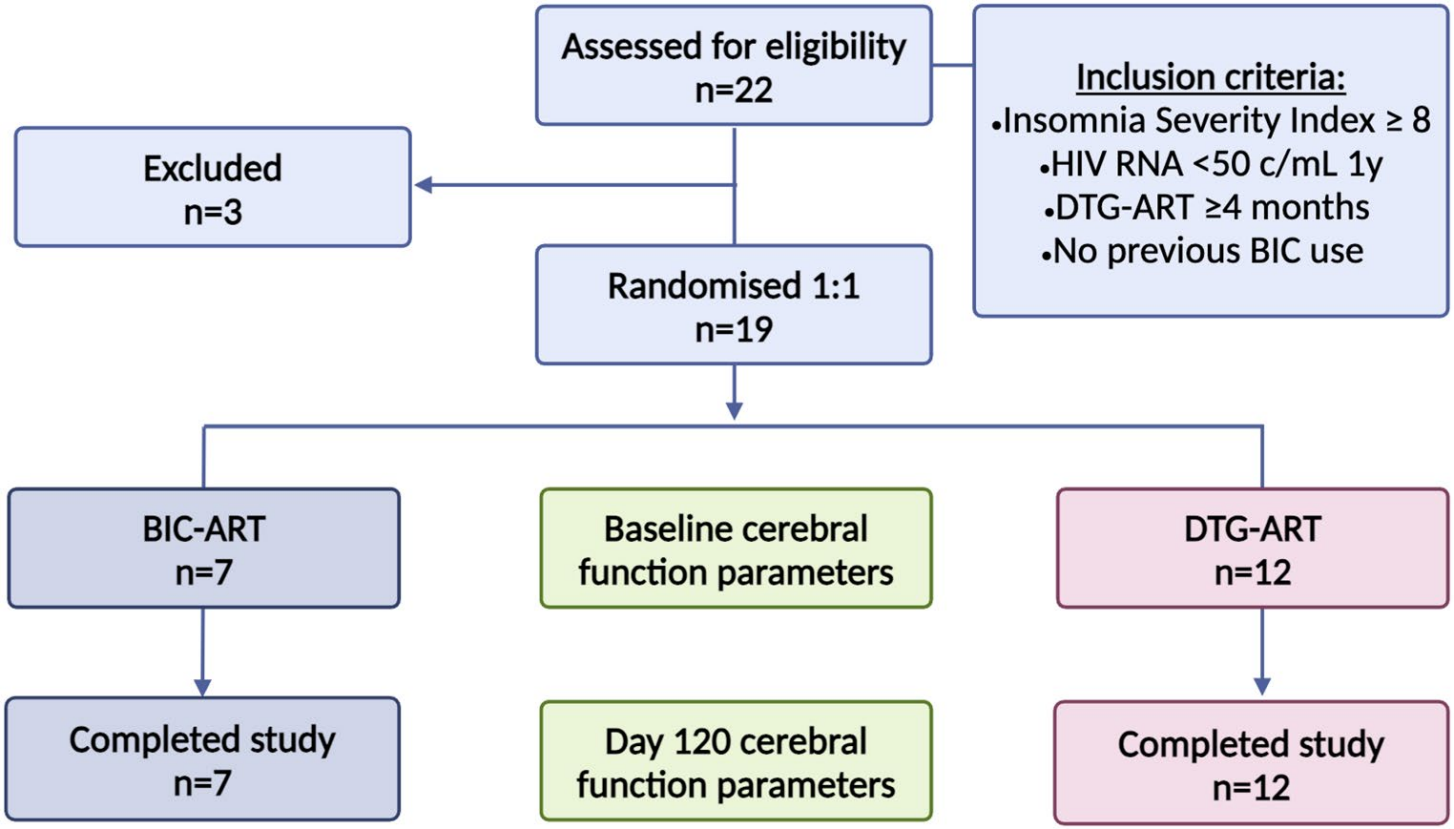
BIC CNS Study design and procedures. Abbreviations: BIC-ART (Bictegravir-containing antiretroviral therapy), c/mL (copies/mL), DTG-ART (dolutegravir-containing antiretroviral therapy). Created using biorender. Tregoning, J. (2025) https://BioRender.com/bwpxk53.

At baseline (day 0, D0), participants were randomised on a 1:1 basis to either continue their current DTG-ART regimen or switch to BIC-ART (bictegravir/emtricitabine/tenofovir alafenamide) for 120 days (Fig. 1). Randomisation was stratified by recruitment site, sex at birth, age at screening and participant handedness. Cerebral function parameters were assessed at D0 and day 120 (D120) by cerebral fMRI, patient reported outcomes (PROs), and plasma biomarkers of immune activation and inflammation (Neopterin, C-X-C-motif ligand 10 (CXCL10), and interleukin-6 (IL-6)).

## Cerebral function parameters

### Patient reported outcome measures

Participants completed 9 questionnaires at D0 and D120 which evaluated sleep (Insomnia Severity Index, The Pittsburgh Sleep Quality Index (Buysse et al., 1989), Epworth Sleepiness Scale (Johns 1991) and Fatigue Severity Scale of Sleep Disorders (Krupp et al., 1989)), appetite (Food Cravings Questionnaire-Trait and Food Cravings Questionnaire-State (Meule et al., 2014; Meule 2020; Cepeda-Benito et al., 2000)) and health-related quality of life (Work Productivity and Activity Impairment – Specific Health Problem (Reilly et al., 1993), HIV Symptom Index – Symptoms Distress Module and 36-item Short Form Survey (Justice et al., 2001)). As sleep disturbances have been associated with appetite regulation and risk of obesity (Chaput et al., 2023), the previously mentioned validated food cravings questionnaires were included to explore whether insomnia symptoms were associated with changes in food cravings. Global total and/or sub-domain scores from each questionnaire were calculated according to pre-existing individual questionnaire scoring systems. Scoring interpretations are detailed in Table 1. Change in total and domain scores from baseline were calculated, with a minus score indicating an improvement in symptoms.

**Table 1:** Baseline participants characteristics for the total cohort and according to randomised drug arm

Baseline characteristics	Total, *n* = 19	BIC-ART, *n* = 7	DTG-ART, *n* = 12
**Demographics**			
Age (years)	55 (28–74)	55 (29–68)	55 (28–74)
Male gender	19 (100)	7 (100)	12 (100)
Ethnicity			
White	17 (89)	7 (100)	10 (83)
Other, mixed	2 (11)	0 (0)	2 (17)
BMI (kg/m^2^)	24 (20–36)	25 (20–36)	23 (20–36)
Ex/current smoker	12 (63)	3 (43)	9 (75)
Recreational drug use	5 (26)	2 (29)	3 (25)
Currently employed	13 (68)	4 (57)	9 (75)
Sexual orientation	*n* = 18	*n* = 7	*n* = 11
MSM	16 (89)	6 (86)	10 (91)
**HIV characteristics**			
Duration of known HIV infection (years)	11 (1–31)	8 (3–19)	11.5 (1–31)
Absolute CD4 count (cells/µL)	605 (318–1133)	620 (512–887)	570 (318–1133)
**ART characteristics**			
Duration on current ART (months)	41 (13–91)	45 (13–76)	49 (14–91)
Baseline ART regimen			
DTG/ABC/3TC	12 (63)	3 (43)	9 (75)
DTG, TDF/FTC	5 (26)	3 (43)	2 (17)
DTG/3TC	2 (11)	1 (14)	1 (8)
**Laboratory characteristics**			
Total cholesterol (mmol/L)	5.0 (3.5–6.8)	3.8 (4.7–5.5)	5.0 (3.5–6.8)

n (%) or median (Q1, Q3) unless otherwise stated. Abbreviations: ABC (Abacavir), ART (Antiretroviral therapy), BIC (Bictegravir), CXCL10 (CXC-Motif Ligand 10), DTG (Dolutegravir), 3TC (Lamivudine), MSM (men having sex with other men), TDF (Tenofovir disoproxil)

## Neuroimaging assessments - resting-state fMRI

### Data acquisition

MRI data was acquired using a 32-channel head coil at two imaging sites: Imperial College London (Siemens 3T Verio MR scanner) and at the University of Sussex (Siemens 3T Prisma MR scanner). The following scans were collected from each participant at D0 and D120: a high resolution 3d fluid-attenuated inversion recovery (FLAIR) sequence (TE = 387ms, TR = 5000ms, field-of-view = 230 × 230mm^2^, 192 contiguous slices of 0.90 mm thickness, voxel size = 0.4 × 0.4 × 0.9mm^3^); a three dimension (3D) magnetisation-prepared rapid gradient-echo (MPRAGE) (TE = 2.01ms, TR = 2300ms, flip-angle = 9°, field-of-view = 256 mm, 160 contiguous slices of 1 mm thickness, voxel size 1mm^3^); and resting state fMRI images were acquired using a BOLD response sensitive echo-planar imaging (EPI) sequence with multi-band acceleration factor = 3 with the following parameters: TE = 28ms, TR = 1520ms, field-of-view = 208 × 208mm^2^, 72 interleaved slices, and voxel size = 2 mm^3^. Participants were instructed to remain still with their eyes closed and not think of anything specific while trying to remain awake. According to protocol, FLAIR scans were reviewed by a neuroradiologist to exclude the presence of significant macroscopic brain abnormalities.

### Data pre-processing

Raw MRI data was converted from DICOM into NIfTI (Neuroimaging Informatics Technology Initiative) format and organised in the Brain Imaging Data Structure (BIDS) format; a standard for organising and describing neuroimaging datasets, in a machine-recognisable format (Gorgolewski et al., 2016), using a heuristic-centric DICOM converter (HeuDiConv version 1.1.6). Anatomical and functional data were pre-processed using a robust pre-processing pipeline with CONN toolbox (V22.a) (Nieto-Castanon 2020b) including realignment with correction of susceptibility distortion interactions, slice timing correction, outlier detection, direct segmentation and Montreal Neurological Institute (MNI)-space normalization, and finally smoothing. Functional data were realigned using Statistical Parametric Mapping (SPM12) toolbox’ realign & unwarp procedure (Andersson et al., 2001), where all scans were co-registered to a reference image (first scan of the first session) using a least squares approach and a 6 parameter (rigid body) transformation (Friston et al., 1995), and resampled using b-spline interpolation to correct for motion and magnetic susceptibility interactions. Temporal misalignment between different slices of the functional data (acquired in interleaved Siemens order) was corrected following SPM slice-timing correction (STC) procedure (Henson et al., 1999; Sladky et al., 2011), using sinc temporal interpolation to resample each slice BOLD timeseries to a common mid-acquisition time. Potential outlier scans were identified using artefact detection tools (ART) (Whitfield-Gabrieli, 2011) as acquisitions with framewise displacement above 0.9 mm or global BOLD signal changes above 5 standard deviations (Nieto-Castanon 2022; Power et al., 2014), and a reference BOLD image was computed for each subject by averaging all scans excluding outliers. Functional and anatomical data were normalized into the MNI (Montreal Neurological Institute) standard space, segmented into grey matter, white matter, and cerebrospinal fluid tissue classes, and resampled to 2 mm isotropic voxels following a direct normalization procedure (Nieto-Castanon 2022; Calhoun et al., 2017) using SPM unified segmentation and normalization algorithm (Ashburner and Friston 2005; Ashburner 2007) with the default IXI-549 tissue probability map template. Last, functional data were smoothed using spatial convolution with a Gaussian kernel of 8 mm full width half maximum (FWHM).

### Denoising

functional data were denoised using the CONN toolbox, following a standard denoising pipeline (Nieto-Castanon 2020a) including the regression of potential confounding effects characterized by white matter timeseries (5 CompCor (*Component-based noise correction*) noise components), CSF timeseries (5 CompCor noise components), motion parameters and their first order derivatives (12 factors) (Friston et al., 1996), outlier scans (below 51 factors) (Power et al., 2014), session and task effects and their first order derivatives (4 factors), and linear trends (2 factors) within each functional run, followed by bandpass frequency filtering of the BOLD timeseries (Hallquist et al., 2013) between 0.008 Hz and 0.09 Hz. CompCor (Behzadi et al., 2007; Chai et al., 2012) noise components within white matter and CSF were estimated by computing the average BOLD signal as well as the largest principal components orthogonal to the BOLD average, motion parameters, and outlier scans within each subject’s eroded segmentation masks. From the number of noise terms included in this denoising strategy, the effective degrees of freedom of the BOLD signal after denoising were estimated to range from 72 to 95.2 (average 90.6) across all subjects (Nieto-Castanon 2022).

### First-level analysis

Seed-based connectivity maps (SBC) and region-to-region (ROI)-to-ROI connectivity matrices were estimated characterising the patterns of functional connectivity with HPC-ICA networks (Desikan et al., 2006) and Harvard-Oxford atlas ROIs (Nieto-Castanon 2020c). We selected 5 regions of interest for fMRI analyses, focused on cognitively relevant networks associated with either sleep (default mode network (DMN) (Horovitz et al., 2009) or dolutegravir-use (dorsal attention network (DAN), sensory motor network (SMN) and associative visual network (VISAS) based on previous pilot studies (Toniolo et al., 2020), as well as an additional network known to be affected by HIV (salience network(Thomas et al., 2013). Functional connectivity strength was represented by Fisher-transformed bivariate correlation coefficients from a weighted general linear model (weighted-GLM) (Nieto-Castanon 2020c), defined separately for each pair of seed and target areas, modelling the association between their BOLD signal time series.

### Second-level analysis

Group-level analyses were performed using a General Linear Model (GLM) (Nieto-Castanon 2020c). For each individual voxel a separate GLM was estimated, with first-level connectivity measures at this voxel as dependent variables (one independent sample per subject and one measurement per experimental condition), and groups or other subject-level identifiers as independent variables. Voxel-level hypotheses were evaluated using multivariate parametric statistics with random-effects across subjects and sample covariance estimation across multiple measurements. Inferences were performed at the level of individual clusters (groups of contiguous voxels). Cluster-level inferences were based on parametric statistics from Gaussian Random Field theory (Nieto-Castanon 2020c; Worsley et al., 1996). Thresholds for results were created using a combination of a cluster-forming *p* < 0.001 voxel-level threshold, and a familywise corrected p-FDR < 0.05 cluster-size threshold (Chumbley et al., 2010).

### Clinical and biomarker assessments

Haematology and biochemistry panels, CD4 + T cell count, and HIV RNA concentrations were measured at days 0, 30 and 120 according to locally approved laboratory protocols. Plasma samples from D0 and D120 were aliquoted and stored at − 80 °C prior to biomarker analysis. Plasma samples were then thawed and analysed according to manufacturer’s instructions. Concentrations of plasma CXCL10 and IL-6 were measured using u-plex^®^ multiplex immunoassays (Meso Scale Diagnostics, Rockville, USA). Neopterin and soluble CD14 (sCD14) were measured using enzyme-linked immunosorbent assays (ELISA; Tecan and Bio-techne (Minneapolis, USA), respectively). Concentrations of plasma biomarkers were measured in duplicate. The rationale for measuring these biomarkers was that neopterin and CXCL10 have previously been associated with cognitive disorders in persons with HIV (Ruhanya et al., 2021; Fleischman et al., 2018), and IL-6 is a marker of systemic inflammation which has been associated with both disease progression and depression in HIV (Grund et al., 2016; Mudra Rakshasa-Loots et al., 2022).

### Statistical analyses

Differences in D0 and D120 PRO change scores and biomarker concentrations between randomised drug arms were analysed using the Mann-Whitney U test. For PROs which demonstrated a significant difference between randomised drug arms in univariate analyses (*p*-values *≤* 0.05) hierarchical linear regression modelling was performed to determine firstly, the impact of randomised drug arm (using BIC-ART as the reference control) (model 1), followed by patient-confirmed recreational drug use (reference control ‘yes’) and a history of anxiety or depression (model 2) (reference control ‘yes’), on variation in domain scoring. Within group analyses of those remaining on DTG-ART were performed using Wilcoxon matched-pair rank testing. Pairwise bivariate correlations between biomarker concentrations were analysed using Spearman’s correlation. Change in resting state fMRI functional connectivity between D0 and D120 were examined, and correlations between connectivity changes, PRO measures and biomarker concentrations determined in those switched to BIC-ART, when compared to those continued on DTG-ART. For all analyses, p-values *≤* 0.05 were considered statistically significant. Statistical analyses and data management were performed using RStudio Version 2024.12.0 + 467, GraphPad Prism version 10.0.2. and CONN toolbox.

## Results

### Baseline participant characteristics

A total of 19 participants were enrolled to the study, of which 7 (37%) were randomised to BIC-ART and 12 (63%) remained on DTG-ART. Baseline participant characteristics are summarised in Table 1. The median age was 55 (28–74) years, all were male and 89% of white ethnicity, with a median CD4 count of 605 (range 318–1133) cells/µL. The median duration of known HIV was 11 (range 1–31) years and duration on current ART was 41 (13–91) months. The majority of individuals (63%) were on dolutegravir/abacavir/lamivudine at baseline. Other baseline ART regimens included dolutegravir, tenofovir disoproxil/emtricitabine (26%) and dolutegravir/lamivudine (11%). Of 19 individuals, 5 (26%) had a history of anxiety or depression (3 (42%) BIC-ART, 2 (17%) DTG-ART). Median PRO scores at baseline are described in Table 2. In line with inclusion criteria all participants had an Insomnia Severity Index Score above 8. The median Insomnia Severity Score for the total cohort at baseline was 15.5 with scores of 15 or more indicating clinically significant symptoms of clinical insomnia.

**Table 2:** Baseline participants reported outcome scores for the total cohort and according to randomised drug arm

Baseline characteristics	Total, *n* = 19	BIC-ART, *n* = 7	DTG-ART, *n* = 12
**Sleep**			
Insomnia severity index	15.5 (8, 26)	14 (11, 21)	17 (8, 26)
Pittsburgh Sleep Quality Index	9 (2, 17)	10 (5, 13)	9 (2, 17)
Epworth Sleepiness Scale	5 (0, 15)	10 (2, 13)	5 (0, 15)
Fatigue Severity Scale	32 (11, 49)	32 (27, 49)	32.5 (11, 48)
**Quality of life**			
Work Productivity and Activity Impairment			
Absenteeism	0 (0, 50)	0 (0, 10)	0 (0, 50)
Presenteeism	30 (0, 70)	55 (40, 70)	20 (0, 70)
Work productivity loss	40 (0, 70)	55 (40, 70)	30 (0, 70)
Activity impairment	30 (0, 80)	65 (30, 80)	30 (0, 70)
HIV Symptom Index (SDM)	12 (0, 61)	12 (0, 27)	11.5 (4, 61)
36-item Short Form Survey			
Physical functioning	95 (60, 100)	95 (60, 100)	100 (85, 100)
Role physical	100 (0, 100)	75 (0, 100)	100 (25, 100)
Role emotional	67 (0,100)	33 (0, 100)	83 (33, 100)
Energy fatigue	53 (25, 90)	45 (25, 80)	57 (30, 90)
Emotional wellbeing	76 (24, 88)	76 (24, 84)	72 (44, 88)
Social functioning	75 (37.5, 100)	62.5 (37.5, 100)	82 (50, 100)
Pain	100 (65, 100)	90 (67.5, 100)	100 (65, 100)
General health	65 (50, 100)	75 (55, 100)	65 (50, 80)
**Appetite**			
Food Cravings Questionnaire-State	35.5 (15, 45)	38 (15, 45)	34 (15, 45)
Food Cravings Questionnaire-Trait	75 (47, 128)	86 (61, 114)	71.5 (47, 128)

n (%) or median (Q1, Q3) unless otherwise stated. Abbreviations: ART (Antiretroviral therapy), BIC (Bictegravir), SDM (Symptoms distress module).

### Effect of antiretroviral therapy on patient reported outcomes

**Fig. 2:**
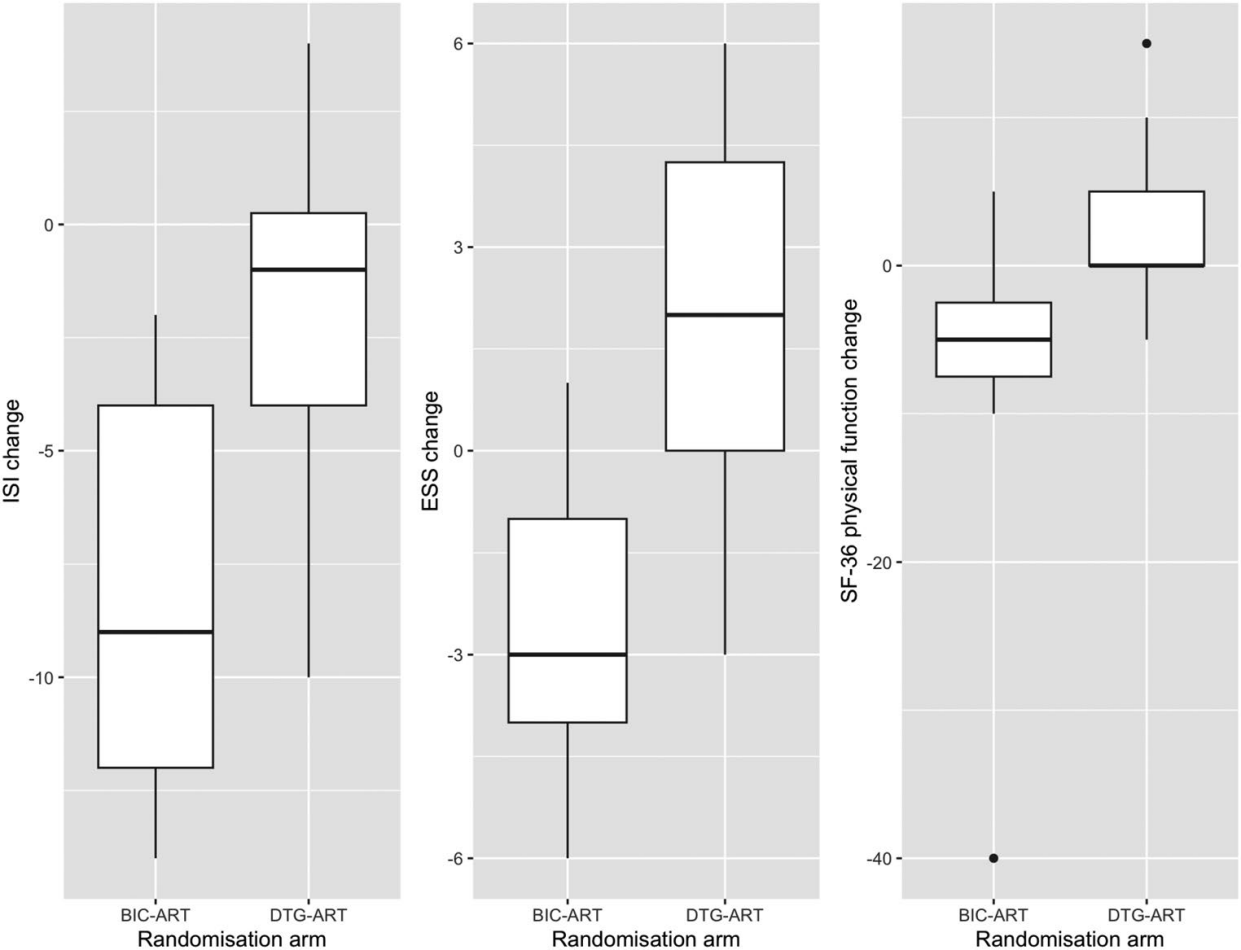
Change in select patient reported outcome measures over 120 days by drug arm. Legend: Randomisation arm shown along the x axis, with ISI (Insomnia Severity Index), ESS (Epworth Sleepiness Scale) and SF-36 (short-form 36) subdomain physical function shown along the y axis. From left to right, the plots demonstrate change in the ISI, ESS and SF-36 subdomain physical function over the 120-day study period according to randomised drug arm. Plots demonstrate median and interquartile range. The PROs shown in these plots were those identified as significant in univariate analyses (ISI *p* = 0.03, ESS *p* = 0.007, SF-36 subdomain physical function *p* = 0.026)

An improvement in symptoms of insomnia (Insomnia Severity Index median change score − 9.0 (range − 14.0, -2.0) vs. -1 (range − 10.0, -4.0), *p* = 0.03) and daytime somnolence (Epworth Sleepiness Scale median change score − 3.0 (range − 6.0, -1.0) vs. 2 (range − 3.0, 6.0), *p* < 0.01) was observed between D0 and D120 in those randomised to BIC-ART, when compared to those continued on DTG-ART. Perceptions of health related to physical functioning also improved from D0 in those randomised to BIC-ART, when compared to those continued on DTG-ART (36-item Short-Form Survey subdomain physical function median change score − 5.0 (-range 40.0, 5.0) vs. 0 (range − 5.0, 15.0), *p* = 0.03) (Fig. 2; Table 4). These observations persisted in regression analyses, which were adjusted for recreational drug use and a history of anxiety or depression (Table 3). No other statistically significant changes in PRO scores were observed by univariate or multivariate analysis (Tables 3 and 4).

**Table 3:** Patient reported outcome change scores over 120 days by drug arm

PRO domain	BIC-ART, *n* = 7	DTG-ART, *n* = 12	*p*-value
**Sleep**	**Median (range)**	**Median (range)**	
Insomnia severity index	-9.0 (-14.0, -2.0)^1^	-1.0 (-10.0, -4.0)	**0.030**
Pittsburgh Sleep Quality Index	-0.0 (-5.0, 1.0)	-0.5 (-3.0, 4.0)	0.413
Epworth Sleepiness Scale	-3.0 (-6.0, 1.0)	-2.0 (-3.0, 6.0)	**0.007**
Fatigue Severity Scale	-10.0 (-16.0 to 8.0)	-1.5 (-23.0 to 13.0)	0.108
**Quality of life**			
Work Productivity and Activity Impairment			
Absenteeism	-0.0 (-10.0, 0.0)^2^	-0.0 (-50.0, 0.0)^3^	0.612
Presenteeism	-0.0 (-40.0, 10.0)^2^	-10.0 (-20.0, 40.0)^4^	0.389
Work productivity loss	-0.0 (-40.0, 10.0)^2^	-10.0 (-40.0, 40.0)^5^	0.938
Activity impairment	-5.0 (-50.0, 10.0) ^2^	-0.0 (-20.0, 40.0)^3^	0.862
HIV Symptom Index (SDI)	-0.0 (-13.0, 12.0)	-1.0 (-54.0, 16.0)	0.866
36-item Short Form Survey			
Physical functioning	-5.0 (-40.0, 5.0)	-0.0 (-5.0, 15.0)	**0.026**
Role physical	-3.0 (-75.0, 25.0)	-0.0 (-99.0, 100.0)	0.117
Role emotional	-33.0 (-67.0, 0.0)	-0.0 (-67.0, 67.0)	0.086
Energy fatigue	-5 (-25.0, 15.0)	-2.5 (-20.0, 10.0)	0.763
Emotional wellbeing	-0.0 (-4, 0.0)	-4.0 (-16.0, 23.0)	0.636
Social functioning	-12.5 (-25.0, 12.5)	-0.0 (-25.0, 25.0)	0.132
Pain	-0.0 (-12.5, 10.0)	-0.0 (-20.0, 20.0)	0.825
General health	-0.0 (-20.0, 5.0)	-2.5 (-30.0, 30.0)	0.416
**Appetite**			
Food Cravings Questionnaire-State	-5.0 (-23.0, 17.0)	-2.0 (-16.0, 23.0)	0.642
Food Cravings Questionnaire-Trait	-8.0 (-21.0, 9.0)	-4.5 (-22.0, 16.0)	0.471

**Table 4:** Hierarchical linear regression analysis of factors associated with change in patient reported outcome measures from baseline

Model	Variable	Beta^1^	95% CI^2^	*p*-value
Insomnia Severity Index				
1	Randomised drug arm (*Ref* BIC-ART)	-6.28	-1.26–11.30	**0.018**
2	Randomised drug arm (*Ref* BIC-ART) Recreational drug use (*Ref* Yes) History of anxiety or depression (*Ref* Yes)	-6.33 -3.13 -0.45	-0.95–11.70 -2.83–9.09 -5.69–6.61	**0.024** 0.277 0.873
**Epworth Sleepiness Scale**				
1	Randomised drug arm (*Ref* BIC-ART)	-4.40	-1.75–7.06	**0.003**
2	Randomised drug arm (*Ref* BIC-ART) Recreational drug use (*Ref* Yes) History of anxiety or depression (*Ref* Yes)	-4.38 -1.96 -0.35	-1.54–7.23 -5.32–1.40 -3.15–3.85	**0.005** 0.232 0.835
**SF36 physical function**				
1	Randomised drug arm (*Ref* BIC-ART)	-11.07	-1.15–20.99	**0.031**
2	Randomised drug arm (*Ref* BIC-ART) Recreational drug use (*Ref* Yes) History of anxiety or depression (*Ref* Yes)	-13.76 -0.79 -10.18	-3.93–23.60 -12.40–10.81 -22.29–1.93	**0.009** 0.886 0.093

### Effect of antiretroviral therapy on resting-state fMRI networks

**Fig. 3:**
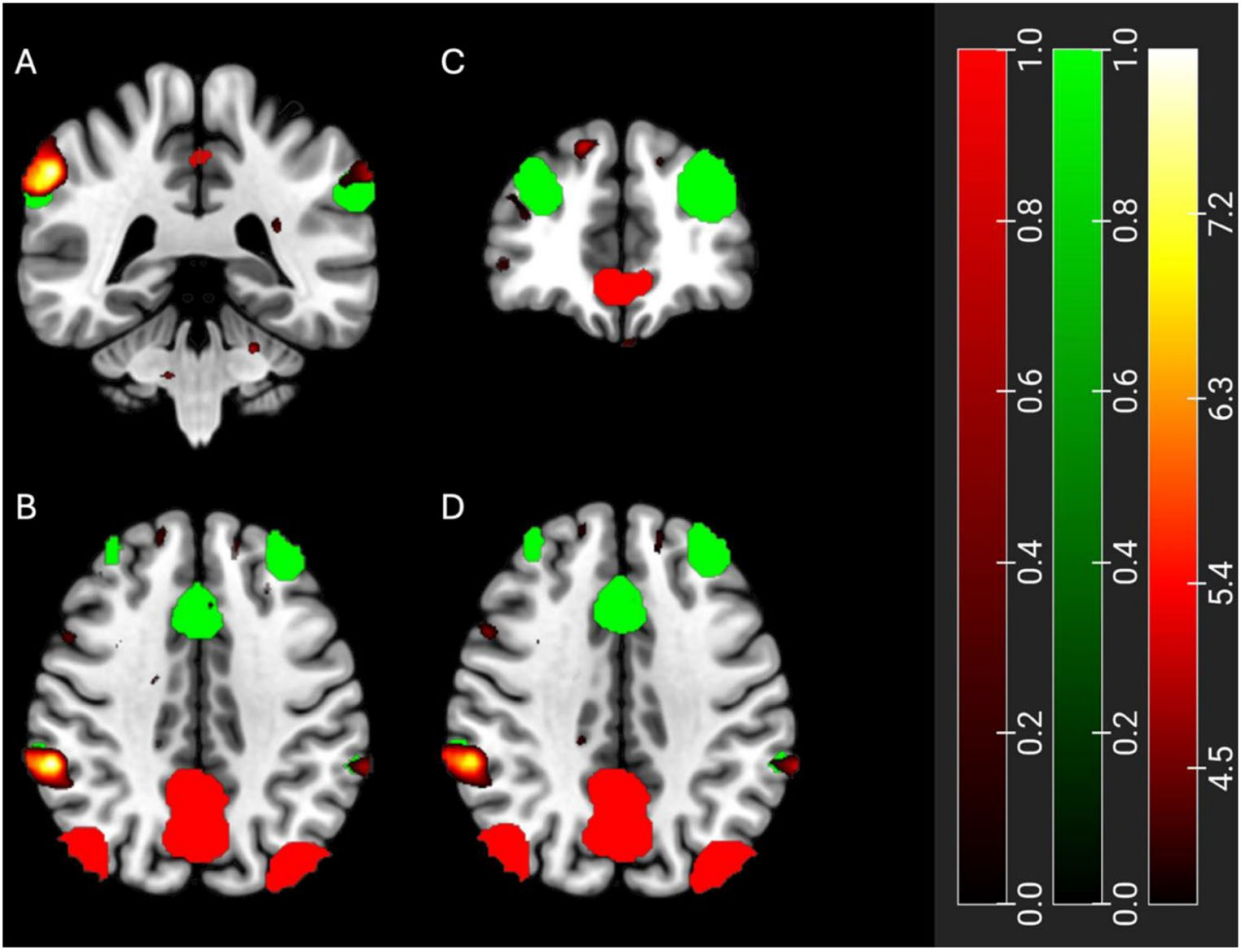
Resting-state functional connectivity after switching to BIC-ART, when compared to DTG-ART. Legend: Locations of the Default Mode Network (DMN) and salience network (SAL) highlighted in red and green, respectively. A and B) Seeds in DMN demonstrating increased functional connectivity with the supramarginal right gyrus (orange) in coronal (**A**) and transverse sections of the brain (**B**). **C** and **D**) Seeds in in the SAL demonstrating increased connectivity with the left and right frontal poles (gradients of orange) in coronal (**C**) and transverse sections of the brain (**D**). Statistical significance was made at the cluster level, utilising cluster-level threshold of corrected p-FDR < 0.05 after applying an uncorrected voxel threshold of *p* < 0.001

Increased functional connectivity was observed in the default mode and salience networks at D120 in those randomised to BIC-ART, when compared to those on DTG-ART: seeds in default mode network (DMN) (increased connectivity with the supramarginal right gyrus; Fig. 3) and in the salience network (SAL) (increased connectivity with the left and right frontal poles; Fig. 3) (cluster-forming *p* < 0.001 voxel-level threshold, corrected p-FDR < 0.05 cluster-size threshold). We found no other differences in brain connectivity between groups over the study period in any of the other three networks examined, which included the dorsal attention, associative visual and sensorimotor networks.

Positive correlations were observed between increased functional connectivity within the DMN and change in PRO measures of sleep (Epworth Sleepiness Scale) and quality of life (36-item Short-Form Survey subdomain physical function) (cluster-forming *p* < 0.001 voxel-level threshold, corrected p-FDR < 0.05 cluster-size threshold) in those switched to BIC-ART, when compared to those continued on DTG-ART. No correlations were observed between the SAL network and PRO measures.

### Effect of antiretroviral therapy on longitudinal biomarker measurements

Of the 19 individuals enrolled to the study, 17 had stored plasma samples at D0 and D120 available for biomarker analysis. Median biomarker concentrations at baseline Neopterin (*n* = 18) 5.06 nmol/L (IQR 4.16, 6.65), CXCL10 559.10 pg/mL (IQR 482, 781) and IL-6 3.07 pg/mL (1.37, 4.78). Change in biomarker concentrations did not differ significantly longitudinally within group (Table 5) or by treatment arm (Table 6). No correlations were observed with biomarker concentrations and any of the neuroimaging parameters. Positive bivariate correlations were identified between baseline plasma IL-6 and CXCL10 concentrations (ρ = 0.61, *p* = 0.014)).

**Table 5:** Change in biomarker concentrations between day 0 and 120

Biomarker	Day 0		Day 120			
	Median (IQR)	*N*	Median (IQR)	*N*	Mean change	*p*-value^2^
Neopterin (nmol/L)	5.06 (4.16, 6.65)	18	6.34 (4.78, 7.56)	18	1.44	0.13
CXCL10 (pg/mL)	559 (482, 781)	17	606 (542, 751)	17	-38.33	0.93
IL-6 (pg/mL)	3.07 (1.37, 4.78)	17	0.64 (0.64, 2.05)	17	-0.97	0.28

1CI (Confidence Interval), 2Mann-Whitney U test. Abbreviations: CXCL10 (C-X-C motif chemokine 10), IL-6 (Interleukin 6), IQR (interquartile range).

**Table 6:** Median change in biomarker concentrations over 120 according to randomised drug arm

Biomarker	BIC-ART		DTG-ART		
	Median (IQR)	*N*	Median (IQR)	*N*	*p*-value^2^
Neopterin (nmol/L)	0.28 (-1.29, 0.81)	6	0.91 (-0.25, 2.11)	11	0.22
CXCL10 (pg/mL)	-94.6 (-145, -4.79)	5	23.2 (-157, 164)	11	0.14
IL-6 (pg/mL)	-2.80(-3.64, -2.44)	5	0 (-2.44, 1.10)	11	0.59

1CI (Confidence Interval), 2Mann-Whitney U test. Abbreviations: CXCL10 (C-X-C motif chemokine 10), IL-6 (Interleukin 6), IQR (interquartile range).

## Discussion

To our knowledge, this pilot study was the first longitudinal, randomised controlled trial to compare the effect of switching INSTIs from DTG- to BIC-ART on cerebral function parameters including resting state fMRI, in persons with HIV with viral suppression and with insomnia. Over the 120-day study period we observed an improvement in self-reported outcome measures of sleep and health-related quality of life, which correlated with increased functional connectivity within resting state fMRI networks associated with sleep in those randomised to BIC-ART, when compared to DTG-ART; findings which may support the use of BIC-ART in persons with HIV with insomnia unable to tolerate DTG-ART.

PRO measures of sleep (Insomnia Severity Index and Epworth Sleepiness Scale) and health-related quality of life (36-item Short-Form Survey subdomain physical function) improved following switch to BIC-ART, when compared to those continued on DTG-ART. While comparable scores were observed between randomisation arms at baseline for the Insomnia Severity Index and the 36-item Short-Form Survey subdomain physical function, those in the BIC-ART arm had higher scores for the Epworth Sleepiness Scale (median baseline score 10 vs. 5, respectively). However, these differences are unlikely to be of clinical relevance given scoring interpretations suggested normal levels of daytime sleepiness (Epworth Sleepiness Scale scores below 10) within both groups (Johns 1991). Further, participants had *symptomatic* sleep disturbance at study enrolment which improved with the switch to BIC-ART. Despite the subjective nature of PRO measures and the open-label study design, which may have introduced the potential for bias, these findings may represent a genuine treatment effect, rather than the effect of repeated measures or the perceived benefit of ART-switch alone. Irrespective of the mechanisms underlying these improved symptoms, importantly, participants switching to BIC-ART experienced benefits to both sleep and quality of life. Following completion of the trial, all 7 participants in the DTG-ART were offered the option to switch to BIC-ART.

Advanced imaging techniques such as fMRI offer a sensitive method for detecting subtle changes in brain function. Previous cross-sectional data have demonstrated reduced functional connectivity within certain neural networks, such as the default mode, salience, executive control and visual networks in persons with HIV, when compared to persons without HIV (Thomas et al., 2013; Wang et al., 2011). These effects may be partially ameliorated with ART, with similar functional connectivity observed in certain neural networks in those with HIV on ART, when compared to those without HIV (Ortega et al., 2015, Thippabhotla et al., 2023). However, limited longitudinal data exists on RS fMRI networks in persons with HIV on ART. Zhang and colleagues demonstrated an increase in cognitive performance and functional connectivity within the DMN 12 weeks after initiating ART (Zhuang et al., 2017). Toniolo and colleagues demonstrated improved functional connectivity within specific neural networks 4 months after switching ART from either efavirenz- to rilpilvirine-based ART (dorsal attention network), or raltegravir-to dolutegravir-based ART (dorsal attention, sensorimotor and associative visual networks); findings which suggest that different ART regimens may have differential effects on functional connectivity (Toniolo et al., 2020). Adding to these data, we observed an increase in functional connectivity within the default mode and salience networks in those switched to BIC-ART, when compared to those continued on DTG-ART. The DMN is located across a number of brain regions and has several functions related to cognition, demonstrating increased activity during internal thought, such as mind wandering (Mason et al., 2007) and episodic memory (Cabeza et al., 2002). Reduced functional connectivity within the DMN has been demonstrated in individuals with sleep deprivation (Gujar et al., 2010) and insomnia disorder (Dai et al., 2015), as well as other psychiatric and cognitive disorders (Mohan et al., 2016). The salience network functions to switch from internal thought, to focus on external task-related stimuli, mediated by the frontoparietal network (Schimmelpfennig et al., 2023). Dysfunction of the salience network may be associated with a number of neuropsychiatric disorders from anxiety (overactivity) to schizophrenia and bipolar (underactivity) (Schimmelpfennig et al., 2023). We observed a correlation between increased functional connectivity in the DMN and improved PRO measures of sleep in those switched to BIC-ART, suggesting that BIC-ART may have an impact on brain regions related to sleep. However, the mechanisms underlying these effects remain unclear and further work to evaluate these associations is warranted.

Sleep disturbances remain prevalent in persons with HIV despite viral suppression and may significantly impact on quality of life. Kunisaki and colleagues recently examined the prevalence of sleep disorders in 357 persons with HIV and 126 lifestyle-similar individuals without HIV. A total of 21% of individuals met the criteria for insomnia, compared with 5% of those without HIV, which was associated with poorer health related quality of life; findings which are supported in the literature(Milinkovic et al., 2020). While the impact of sleep on quality of life is perhaps unsurprising, other adverse health-related outcomes have been reported in association with sleep disturbances, such as reduced cognitive function(De Francesco et al., 2021; Mahmood et al., 2018). Several risk factors have been suggested for the increased risk of sleep disturbances in persons with HIV. Firstly, psychological and behavioural factors which may impact upon sleep, such as depression and anxiety, have a higher prevalence in persons with HIV, when compared to the general population (Gooden et al., 2022). Secondly, in an ageing population with HIV, the increasing prevalence of multimorbidity and polypharmacy may increase the risk of drug-drug interactions and neurotoxic side effects (Henderson and Winston 2025). Lastly, despite modern ART, previous data have demonstrated persistent immune activation and neuroinflammation in virologically suppressed individuals (Vera et al., 2016), which may interfere with normal sleep(Kunisaki et al., 2021). Reassuringly, in our cohort plasma biomarkers of immune activation and neuroinflammation did not significantly change over the follow up period, despite subjective and objective improvements in measures of sleep, suggesting that the underlying mechanisms may not be driven by neuroinflammation.

While CNS side effects related to INSTIs are well recognised, the causative mechanisms behind these effects remain unclear. Initial data suggests that these effects are not a dose-dependent toxicity. The VIKING-3 study examined the safety and efficacy of twice daily dolutegravir containing-ART regimen in treatment-experienced persons with HIV failing current therapy. Despite twice daily dosing, discontinuation due to adverse events remained low (3%); however, it should be acknowledged that this cohort included individuals with relatively advanced HIV and the low frequency of adverse events may have represented a clinical improvement while on suppressive ART, rather than an absence of CNS side effects (Castagna et al., 2014). Other studies have highlighted a potential association between specific genetic variants related to drug metabolism and neuropsychiatric side effects in persons with HIV on dolutegravir (Yagura et al., 2017; Borghetti et al., 2019). Further, a higher risk of dolutegravir-related neuropsychiatric side effects has been observed when co-administered with abacavir, in women and in those over the age of 60 years (Hoffmann et al., 2017; Cid-Silva et al., 2017). At baseline *n* = 12 (63%) of our study population were on ART regimens containing both dolutegravir and abacavir. Due to the small numbers of participants, we were unable to make observations with regards to the association of ART regimens including dolutegravir and abacavir, on sleep.

Strengths of this study were the inclusion of individuals with a suppressed viral load, which reduced the potential confounder of unsuppressed viraemia on fMRI networks. The use of fMRI in symptomatic individuals as well as those with symptoms also helps to provides data on potential neural networks affected by sleep disturbances. Further, adding strength to our findings, we did not observe a change in any of the observed cerebral function parameters in those who remained on DTG-ART. While the observed improvement in PROs and resting state fMRI networks associated with sleep and QoL with BIC-ART may partly reflect the effects of withdrawal from DTG-ART, rather than the benefit of BIC-ART alone, the 120-day follow-up period on BIC-ART helped to mitigate against this risk. Our study was limited by the small sample size which was initially planned for 46 individuals. However, in the period post the COVID-19 mobility restrictions, we were unable to meet our recruitment target. Despite 1:1 randomisation, due to recruitment challenges there was also an unequal distribution of participants between study arms. The estimated sample required to detect a difference in a ROI, the insula, that had the biggest effect size from our pilot data (Toniolo et al., 2020), was 40 participants (20 per treatment arm), for a power of 85% (the standard for fMRI analyses). The small sample size overall and between randomisation arms reduced the power of our observations and results of this study should be therefore considered as exploratory. The study population comprised of predominantly white men, and other groups such as women were not represented, limiting the generalisability of these findings. We lacked data on cognitive performance and so we were unable to determine whether improved functional connectivity correlated with change in cognition.

Despite these limitations, our findings have several clinical implications. Firstly, for individuals with sleep disturbances on DTG-ART, switching to BIC-ART may offer an alternative, robust treatment option with the *potential* for less neuropsychiatric side effects affecting sleep. Secondly, the observed correlation between subjective measures of sleep with changes in resting state functional MRI networks associated with sleep, highlights the potential utility of fMRI in both the assessment of sleep disorders in persons with HIV, and in assessing the impact of ART on the CNS.

## Conclusions

In this exploratory study of individuals with well controlled HIV and symptoms of insomnia on DTG-ART, those who switched to BIC-ART had improvements in self-reported measures of sleep and QoL, which correlated with increased functional connectivity in resting state fMRI networks associated with sleep, when compared to those continued on DTG-ART. In light of the small numbers in this study, these observations should be interpreted with caution and further validation of these findings in larger scale clinical trials is warranted.

## Data Availability

No datasets were generated or analysed during the current study.
